# Evaluation of AgNORs in Oral Potentially Malignant Lesions

**DOI:** 10.1155/2015/218280

**Published:** 2015-08-31

**Authors:** Karin Berria Tomazelli, Filipe Modolo, Elena Riet Correa Rivero

**Affiliations:** ^1^Post-Graduate Program in Dentistry, Federal University of Santa Catarina, 88040-370 Florianópolis, SC, Brazil; ^2^Department of Pathology, Health Sciences Center, Federal University of Santa Catarina, University Campus, Trindade, 88040-370 Florianópolis, SC, Brazil

## Abstract

Oral squamous cell carcinoma (OSCC) is usually preceded by detectable mucosal changes, as leukoplakias and erythroplakia. Histologically, these lesions can range from hyperkeratosis and acanthosis to epithelial dysplasia and even OSCC. The aim of this study was to investigate the proliferative activity, using AgNORs quantification proteins, in low- and high-risk oral epithelial dysplasia, OSCC, and nondysplastic epithelium (inflammatory fibrous hyperplasia). The sample was divided into 4 groups: G1: 10 cases of inflammatory fibrous hyperplasia (IFH), G2: 11 cases of low-risk epithelial dysplasia (LD), G3: 10 cases of high-risk epithelial dysplasia (HD), and G4: 11 cases of OSCC. The quantitative analysis was performed using an image processing software in photomicrographs at 1000x magnification. The one-way ANOVA was used for comparison of the mean AgNORs counts between the study groups. The mean AgNORs count was significantly higher (*P* ≤ 0.01) in OSCC when compared to IFH and the LD; however, it was not statistically different from HD. The mean number of LD was significantly lower than the HD and OSCC, with no difference related to IFH. AgNORs quantification can be an important and cheap method to help in the determination of the degree of epithelial dysplasia and, consequently, in the analysis of their potential for malignant transformation.

## 1. Introduction

Squamous cell carcinoma is the most common malignant tumor of the oral cavity. The survival rate for a patient with oral cancer is low, varying between different ethnicities and age groups [1, 2]; therefore, early diagnosis is essential to improve the treatment of this condition [2]. Most oral squamous cell carcinomas (OSCCs) develop from potentially malignant lesions and are clinically present as leukoplakia, erythroplakia, or erythroleukoplakia [1].

Leukoplakia is a clinical term for a lesion defined as a white patch or plaque that cannot be removed and cannot be characterized clinically or microscopically as any other definable disease [3]. These conditions usually present similar clinical appearances, but microscopically there is a considerable degree of heterogeneity between them [1, 3].

Histologically, leukoplakias may have a wide range of phenotypes such as hyperkeratosis and acanthosis with presence or not of epithelial dysplasia (ED),* in situ* carcinoma, or invasive OSCC. When ED is present, it indicates an abnormal epithelium and disordered growth [1, 3].

Grading ED is still very controversial and involves great subjectivity [4, 5]. To objectify and reduce these problems, the binary system of grading was created, which groups the ED in high risk of malignancy (HD) and low risk of malignancy (LD) [5].

It is believed that most OSCCs are preceded by ED. The probability of malignant transformation increases when the epithelium shows severe changes [1]. Therefore, evaluating the degree of dysplasia is important to predict the potential for malignant transformation of ED and determine the prognosis and treatment [5]. In association with the degree of dysplasia, several biological markers have been investigated in order to predict the progression to cancer [6–8]. Nonetheless, these markers have not gained any use in routine diagnosis and their utility in the prediction of risk of malignant transformation remains unacknowledged.

In this context, the biological behavior of several injuries and/or tumors can be determined by cell proliferation, which is defined as an increase in the number of cells entering the cell cycle [9]. Several biological markers have been used to evaluate cell proliferation such as the cytochemical technique of AgNORs (argyrophilic nucleolar organizer regions) staining [10].

AgNORs staining consists of detecting specific proteins associated with transcriptional activity of the nucleolar organizer regions (NORs) by impregnation of colloidal silver [10]. NORs are loops of DNA that contain ribosomal genes which synthesize the 18S and 28S portions of the ribosomal RNA (rRNA) [11]. These regions correspond to secondary constrictions of metaphase chromosomes of eukaryotic cells which in humans are located on the short arms of chromosomes 13, 14, 15, 21, and 22 [12]. NORs contain a set of acidic proteins, non-histone, which bind the silver ions, thus selectively visualized by silver-staining methods in routine histological samples. For this reason, they are called argyrophilic nucleolar proteins, AgNORs. In the light microscope, NORs can be identified as well-defined black dots located throughout the cell nucleus [10].

The aim of this study was to evaluate the proliferative activity, through the AgNOR count in the oral dysplastic epithelium, nondysplastic epithelium, and OSCC, in order to verify the usefulness of this technique in the prediction of the clinical behavior of epithelial dysplasia.

## 2. Materials and Methods

This study was approved by the Committee of Ethics in Research with Humans at the Federal University of Santa Catarina (UFSC) under number 2271.

### 2.1. Selection of Cases and Morphological Analysis

A retrospective investigation was performed from the reports of biopsy cases sent to the Oral Pathology Laboratory (LPB) of the Federal University of Santa Catarina (UFSC), whose clinical diagnosis was leukoplakia, erythroplakia, or erythroleukoplakia. A survey of cases diagnosed histologically, such as OSCC and inflammatory fibrous hyperplasia (IFH), through histopathological reports was also performed.

The histopathological specimens stained in hematoxylin and eosin (H&E) where selected for evaluation by optical microscopy by two examiners and rated into four groups: Group 1 (G1): 10 cases of IFH, nondysplastic epithelium; Group 2 (G2): 11 cases of low-risk epithelial dysplasia (LD); Group 3: 10 cases of high-risk epithelial dysplasia (HD); and Group 4: 11 cases of SCC. The ED classification was performed according to the new binary system of grading (low risk and high risk of malignancy) [5] by a calibrated examiner, first independently and later by consensus with an oral pathologist.

### 2.2. AgNORs Staining Technique

Formalin-fixed paraffin-embedded tissues were submitted to 3 *μ*m thickness sections and extended in glass slides previously prepared with 3-aminopropyltriethoxysilane (Sigma Chemical Co., St. Louis, MO USA). The sections were dewaxed in xylene and hydrated through decreasing grades of ethanol. The silver staining was applied according to the method of Ploton et al. [10] modified by Rivero et al. [13].

### 2.3. AgNOR Quantification

**Figure 1:**
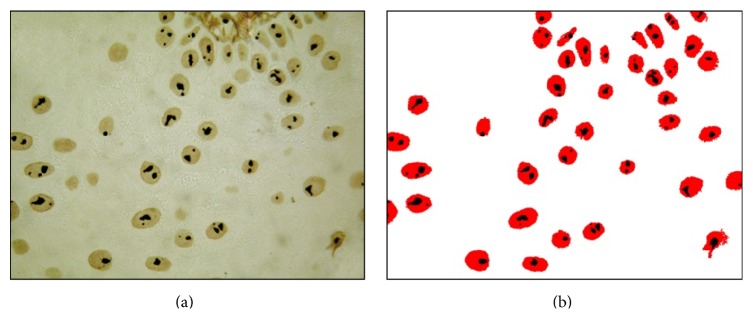
(a) AgNOR staining (1000x); (b) same picture after image processing by the software “counting cells.”

AgNORs quantification was performed in photomicrographs at 1000x magnification, using a “counting cells” software developed by Ferreira et al. [14] (Figure 1). For each case, eight to ten different fields were analyzed, totaling at least 100 nuclei per case. The selection of representative fields for each group was previously performed by microscopic evaluation in H&E.

### 2.4. Statistical Analysis

One-way ANOVA was used to compare the groups. The mean AgNOR count was compared in each group with the three other groups by a post hoc Tukey test in order to confirm the difference among the groups. Differences at *P* < 0.01 were considered significant.

## 3. Results

**Figure 2:**
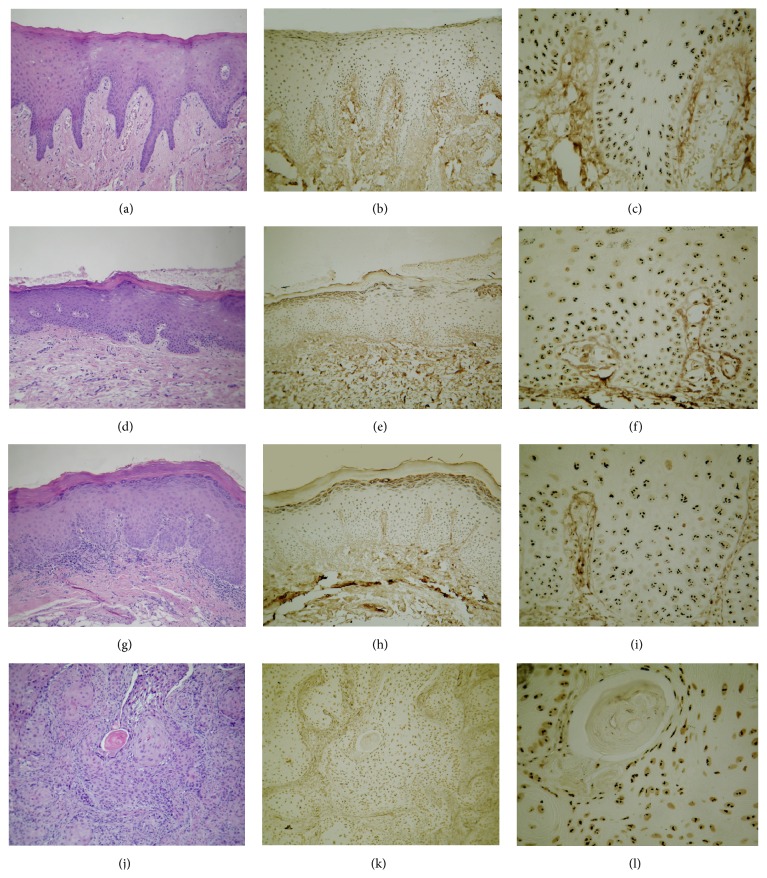
(a), (b), and (c): epithelial lining of inflammatory fibrous hyperplasia; (d), (e), and (f): low risk of malignancy epithelial dysplasia; (g), (h), and (i): high risk of malignancy epithelial dysplasia; (j), (k), and (l): squamous cell carcinoma. (a), (d), (g), and (j): H&E, 100x; (b), (e), (h), and (k): AgNOR, 100x; (c), (f), (i), and (l): AgNOR, 400x.

For all cases of IFH, LD, HD, and OSCC, the AgNORs were visualized in a light microscope as black or brown dots, of distinct sizes round shape with regular boundaries, distributed within the nucleus of the epithelial cells (Figure 2).

The mean AgNOR count showed significant differences between the OSCC (2.73 ± 0.64), as well as HD (2.3 ± 0.48), when compared with the IFH (1.5 ± 0.52) and the LD (1.09 ± 0.3). No statistical difference was observed between the OSCC and HD, as well as between the IFH and LD (Table 1).

## 4. Discussion

According to the World Health Organization (WHO), ED can be defined as mild, moderate, or severe or as carcinoma* in situ*, based on the presence and degree of cellular atypia and architectural changes in the epithelium lining [4]. However, this classification is based on histological criteria, which involves an important subjective component. The diagnosis depends on the emphasis which is put on each of these characteristics for grading by pathologists and, for that reason, is a controversial subject [5, 15, 16]. Thus, to make a more accurate graduation of ED, the binary system of grading has been an alternative, which proposes a new scheme based on the same morphological criteria used by the WHO classification 2005 (architecture and cytology changes), but is based on scoring the features that grade the lesions into either “low risk” or “high risk.” This method makes histological grading reproducible and a good prognosticator for malignant transformation [5].

Whereas the accuracy of grading ED is dependent on the quality of tissues, the site from which the biopsy is taken, and the subjectivity of histological evaluation, several biological markers have been investigated in order to help in the prediction of cancer progression. Among these markers, the following are highlighted: DNA ploidy analysis, loss of heterozygosity, matrix metalloproteinases, and proliferation and differentiation markers [6–8]. Although these markers have contributed greatly to the understanding of the oral cancer progression, until this moment they are still underutilized, because many of these methodologies, as the verification of the DNA ploidy, require specific equipment and additional resources that most diagnostic laboratories could not afford to pay.

AgNORs count is used as a marker of cellular proliferation [17] and for this reason it has been shown as an important tool to characterize the ED in leukoplakia and also OSCC [16, 18–20]. In this study, a significant difference in the means of NORs/nucleus between LD and HD was found, confirming that the AgNOR count can be effective for this distinction [20].

Another important marker for predicting the proliferative activity among benign, premalignant, and malignant oral lesions is the Ki-67 immunohistochemical marker [21, 22]. In Teresa et al. [23], a comparison between the AgNOR histochemical marker and the Ki-67 immunolabeling showed that the AgNOR method clearly discriminated the proliferative status of benign, premalignant, and malignant oral lesions. In addition, Ki-67 did not present statistical significance between dysplastic and nondysplastic leukoplakia, suggesting that Ki-67 could not be used to determine the small difference in cell activity of these lesions. In the present study, we decided to use AgNOR as a proliferation marker, since it is cheaper and simpler than the immunohistochemical technique.

Some authors have shown that the number of NORs/nucleus increases from the ED to OSCC [15, 16, 18, 20, 24, 25]. Considering the WHO classification of dysplasia, an increasing of indexes of AgNORs from lesions with lower degrees of dysplasia to lesions with more severe dysplasia [26] has been demonstrated. On the other hand, Spolidorio et al. [19] found no difference in the AgNORs count between the ED and OSCC; however, a distinction was found in the AgNORs morphology based on their size, shape, and distribution pattern within the nucleus. Although various mean AgNOR related parameters (count, area, perimeter, and proportion) can be used to compare normal oral epithelium from dysplastic and nondysplastic leukoplakia [24], according to Garg et al. [27], the AgNOR count is the most appropriate marker to differentiate between dysplastic and nondysplastic leukoplakia.

In our study, there was no difference between the AgNORs means in the nondysplastic epithelium (IFH lining) and the LD. However, these two groups showed an AgNORs means lower than in the OSCC. Other authors have demonstrated the difference in the AgNORs count in the nondysplastic epithelium and OSCC [18, 28], which can be explained considering that, histologically, the IFH shows discreet epithelial changes with low proliferative activity and the OSCC presents alterations in the entire epithelium extension and neoplastic cells with intense proliferative activity [29]. Similarly, the LD presents discrete epithelial alterations with low proliferative activity, expressing low count of AgNORs. Therefore, our results suggest that the AgNORs count can be proportional to the epithelial proliferative activity, confirmed by previous studies [17, 29–31].

Determining a distinction between the HD and OSCC in the initial stage is often not possible, since both have atypical epithelial cells and high proliferative rates [32]. According to Warnakulasuriya [33], in many cases, the HD can only be differentiated from the OSCC by the absence of invasion to the conjunctive tissue. In addition, Chattopadhyay et al. [18] suggested that when the lesion becomes more dysplastic and malignant, the AgNORs count tends to increase. In the same way, Xie et al. [20] showed that the AgNORs counts can predict the progression of dysplastic lesions to the SCC. This information may explain the results of this study concerning the absence of a difference in the means of the NORs/nucleus between the HD and OSCC. However, it is important to consider as a limitation of the present study the number of cases used.

From the point of view of histological classification, our results reveal that the AgNORs counts can be proportional to epithelium proliferative activity since the LD, as well as the IFH, shows less AgNORs counts than the HD and SCC and, consequently, lower rates of cell proliferation. On the other hand, further studies are warranted to use AgNORs counts as a diagnostic method, since, until now, mean AgNOR decision threshold has not been established to provide a definitive and reproducible diagnostic test.

According to our results, the AgNORs count represents a valuable criterion to help the gradation of epithelial dysplasia and, therefore, help in examining their potential for malignant transformation. However, the AgNORs evaluation should not be used as a definitive diagnostic method, but as a complementary one when there are doubts from the histological criteria.

## Figures and Tables

**Table 1:** Mean AgNOR count by case and by group.

G1-IFH (*n* = 10)	G2-LD (*n* = 11)	G3-HD (*n* = 10)	G4-OSCC (*n* = 11)
1,76	1,57	2,22	4,15
1,74	1,94	2,39	3,45
2,37	1,85	2,2	3,35
1,87	1,57	3,62	2,79
2,23	1,81	2,69	3,68
2,03	1,98	3,11	3,59
1,79	1,93	3,34	2,82
2,17	1,86	2,29	3,88
2,02	1,7	2,47	3,44
1,86	1,94	2,23	2,85
—	2,59	—	2,66

1,5 ± 0,52^a^	1,09 ± 0,3^a^	2,3 ± 0,48^b^	2,7 ± 0,64^b^

Values in each group are expressed as the means ± standard deviation. Different letters indicate a statistically significant difference between the groups (*P* < 0.05).
